# Inkjet Printing of Plate Acoustic Wave Devices

**DOI:** 10.3390/s20123349

**Published:** 2020-06-12

**Authors:** Iren Kuznetsova, Andrey Smirnov, Vladimir Anisimkin, Sergey Gubin, Maria Assunta Signore, Luca Francioso, Jun Kondoh, Vladimir Kolesov

**Affiliations:** 1 Kotelnikov Institute of Radio Engineering and Electronics of RAS, 125009 Moscow, Russia; andre-smirnov-v@yandex.ru (A.S.); anis@cplire.ru (V.A.); kvv@cplire.ru (V.K.); 2 Kurnakov Institute of General and Inorganic Chemistry of RAS, 119991 Moscow, Russia; gubin@igic.ras.ru; 3 CNR, Institute for Microelectronics and Microsystems, 73100 Lecce, Italy; mariaassunta.signore@cnr.it (M.A.S.); lucanunzio.francioso@cnr.it (L.F.); 4 Graduate School of Science and Technology, Shizuoka University, Shizuoka 432-8561, Japan; kondoh.jun@shizuoka.ac.jp

**Keywords:** additive technology, inkjet printing, silver nanoparticle ink, interdigital transducers, acoustic wave device

## Abstract

In the paper, the results of production of Ag inkjet printed interdigital transducers to the acoustic delay line based on Y-cut X-propagation direction of lithium niobate plate for the frequency range from 1 to 14 MHz are presented. Additionally, morphological, structural, and electro-physical characteristics of the obtained electrodes were investigated. Mathematical modeling of the excitation of acoustic waves by these electrode structures was carried out. Comparison of the theoretical results with experimental ones showed their qualitative and quantitative coincidences. It was shown that conventional inkjet printing can replace the complex photolithographic method for production of interdigital transducers for acoustic delay lines working up to 14 MHz. The resulting electrode structures make it possible to efficiently excite acoustic waves with a high value of electromechanical coupling coefficient in piezoelectric plates.

## 1. Introduction

In recent years, work has been actively carried out in the field of creating various electrode structures by the direct inkjet printing [1]. These structures are used to produce thin-film solar panels, radio frequency identification tags, sensors, antennas, and batteries [2,3,4,5,6,7]. It should be noted that inkjet printing can be performed on both rigid and flexible substrates [1,8,9,10]. In addition, this method is allowed to produce 3D structures and electronic components of a large area [10,11,12]. Another advantage of the inkjet printing is the ability to perform it at room temperature. 

For inkjet printing, inks with graphene or colloidal nanoparticles [7,8,9], the inks based on Au, Ag, and Cu nanoparticles [13,14,15,16], were used as printing materials. The inks based on Ag nanoparticles are the most promising. This is due to the resistance of Ag to oxidation, good electrical conductivity, and low cost. In general, the ink contains the particles of the corresponding metal with a size of less than 100 nm, which are dispersed in an aqueous or organic solution with a volumetric content of particles from 20% to 70%. In order to avoid agglomeration, which leads to clogging of nozzles and poor-quality printing, the nanoparticles are stabilized by amphiphilic molecules of polyacrylic acid (PAA) or polyvinylpyrrolidone (PVP) [16,17,18,19]. This leads to electrostatic repulsion of the particles and prevents them from the agglomeration. After printing and drying the solvent, all of the above inks require annealing. This is due to the fact that, although the ink contains metal nanoparticles, they are not conductive, since stabilizing agents create insulating ligand shells that separate particles from each other. 

In contrast to the traditional photolithographic technology, inkjet printing does not require expensive equipment, a clean room, and a large number of technological operations. In addition, this method is very environmentally friendly [20]. 

As is very well known, interdigital transducers (IDT) are the main element of acoustoelectronic devices [21]. The production of the surface acoustic wave (SAW) device with a working frequency of 96 MHz and a wavelength of 40 µm by using a super inkjet printing was recently reported. In this case, the strip width was about 10 µm. This technology requires the use of a specialized expensive superfine inkjet (SIJ) printer [22]. It should be noted that the acoustic waves of zero and higher order in piezoelectric plates have been actively investigated recently to develop various biological, chemical, and physical sensors on their basis [23,24,25,26,27,28,29]. The operating frequency of such sensors is up to 20 MHz. In this case, the width of the strips and the gaps between them in the IDT is hundreds of microns. Thus, there is no need to use super inkjet printing with high resolution to produce acoustic sensors based on the plate acoustic waves. The possibility of the production of a flexible gravimetric sensor based on a polyvinylidene fluoride (PVDF) piezoelectric film with the interdigital transducers created by means of the direct inkjet printing was previously demonstrated [30]. In this case, wavelength was equal to 0.8 mm, and the strip width was equal to 0.2 mm. Moreover, there are known works on the production of an electrode structure by means of inkjet technology and Ag ink of a SAW filter and radio frequency (RF) tags [31,32].

In this paper, we present the results of the production of an acoustic delay line based on Y-cut X-propagation direction (YX) of lithium niobate plate for the frequency range from 1 to 14 MHz by direct inkjet printing. The results of the analysis of morphology, structural characteristics, and resistance of the obtained electrodes are presented. The mathematical modeling of the excitation and the reception of the acoustic waves by the created IDTs was carried out. Comparison of the theoretical results with experimental ones showed their qualitative and quantitative coincidences. It was shown that conventional inkjet printing can replace the complex photolithographic method for production of interdigital transducers for acoustic delay lines and electrode structures for acoustic devices operating at very low frequencies (max few kHz). The resulting electrode structures make it possible to efficiently excite acoustic waves with a high value of electromechanical coupling coefficient in piezoelectric plates. 

## 2. Materials and Methods

### 2.1. Inkjet Printer and Materials

A Dimatix DMP-2831 printer (Fujifilm, Santa Clara, CA, USA) was used for direct inkjet printing. The printer contains a cartridge with a piezo head equipped by 16 nozzles. The volume of output droplet was 10 pl. The printer is also equipped with a three-axis positioning system for the print head, a video monitoring system for the printing process, a vacuum table, and a substrate holder. The substrate thickness supported for printing was laid in the range from 0.5 to 25 mm, and the substrate area was up to 210 mm × 260 mm. The sample stage accuracy of the Dimatix DMP-2831 printer was 30 μm. The production of printed templates was carried out by means of the software package supplied by the manufacturer. A monodisperse silver nano-ink (LLC AkKo Lab, Moscow, Russia) was used. 

**Figure 1:**
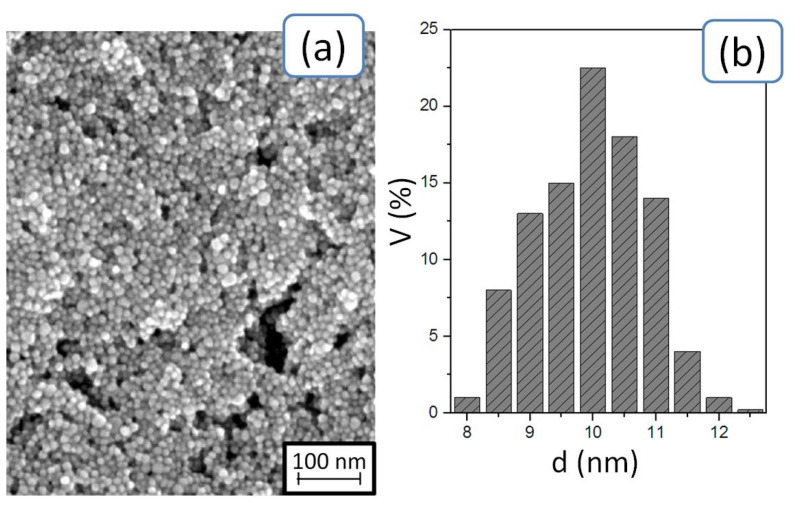
SEM image of Ag nanoparticles (**a**) and a histogram of the particle size distribution (**b**).

An SEM image of Ag nanoparticles (a) and a histogram of the particle size distribution (b) are presented in Figure 1. The size of silver nanoparticles was about 10 ± 2 nm. The content of silver nanoparticles was 10–20 w.t.%, and the ink viscosity was 15–25 µPa·s [33]. 

The electrode structures were deposited on 380 μm thick YX lithium niobate (LiNbO_3_) wafer (Crystal Technology Inc., West Chester, PA, USA). Si wafer 525 μm thick was used as reference material for investigation of the influence of incineration temperature on the morphology and the resistivity of the inkjet printed electrodes. 

### 2.2. Samples Preparation

**Figure 2:**
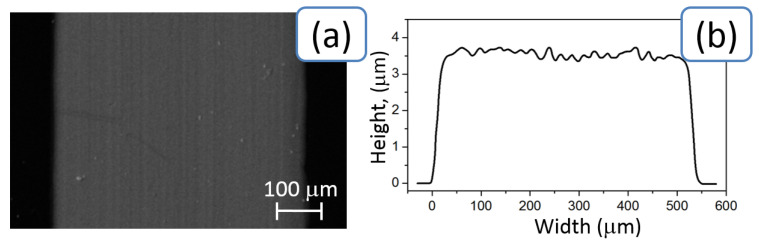
SEM image of the printed Ag line (**a**) and the line profile extracted from atomic force microscopy (AFM) measurement (**b**). The height curve of the conductor over the entire width was average 4 µm.

Si and LiNbO_3_ samples were cleaned by rinsing with ethanol solution and dried at room temperature (24 °C). The conductive strips with widths of 550 μm and lengths of 30 mm were directly inkjet printed onto the polished side of the Si sample. Three to five layers were printed on top of each other in order to increase homogeneity of obtained structures. Figure 2 shows the SEM image (a) and the averaged profile of the obtained strip (b) on the surface of Si. The line profile was measured by means of an atomic force microscopy (AFM) in air with a set up Ntegra Spectra (NT-MDT, Zelenograd, Russia) using Silicon probes NSG-10 with a force constant of 3–37 N/m, a resonance frequency of 140–390 kHz, and a curvature radius of 6 nm. The scanning area was 5 μm × 5 μm. The imaging samples were processed by Gwyddion data analysis software. After printing, the Si samples were annealed in a muffle furnace for 30 min at 100, 200, 300, 400, and 500 °C.

**Figure 3:**
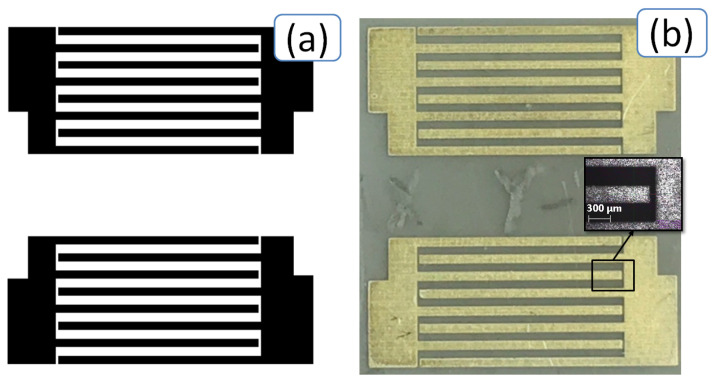
Sketch of the printed layout (**a**) and photo (**b**) of the produced delay line on YX LiNbO_3_ plate. Four pairs of finger electrodes for each IDT were deposited.

The interdigital transducers of the produced plate acoustic wave (PAW) delay line were printed onto the surface of the LiNbO_3_ sample with a width of 13 mm, a length of 14.8 mm, and a thickness of 380 µm. A sketch of the printing layout (a) and a photo (b) of the printed PAW device are presented in Figure 3. The geometric parameters of the sketch of the printing layout are given in Table 1. 

After printing, the LiNbO_3_ samples were dried out at the room temperature for 10 min. Then, they were heated by means of a hot (200 °C) air flow from a Hot Air Rework Soldering Station YH-853AAA (YIHUA, Guangzhou, China). The regime of short pulses (1 s) of hot air flow with it turning off for 5 s was used. 

After this, the gold wires of 25 µm diameter and of 30 mm length were glued to the contact pads by means of conductive glue SilverPrint (GS Electronics, Rockford, IL, USA). S-parameters measurements were performed with a network analyzer E5061B (Keysight, Santa Rosa, CA, USA) operating in phase format. The applied IDT alternating current (AC) voltage was equal to 300 mV.

### 2.3. Electrode Characterization

Morphology of the electrodes printed on Si samples was investigated by means of a scanning electron microscopy (SEM) Mira II (Tescan, Brno-Kohoutovice, Czech Republic) with an operating voltage of 30 kV. SEM images were collected in a secondary electrons mode.

The resistivity of electrodes printed on Si samples was measured by means of a semiconductor device analyzer Agilent B1500A (Keysight, Santa Rosa, CA, USA) in combination with a PM-5 probe station (CascadeMicrotech, Thiendorf, Germany). The measurements were carried out using a pseudo-Kelvin connection (two probes). The measurement range was from −2 to 2 V with a current limit of 30 mA.

The surface tension of Ag nano-ink deposited on the surface of the LiNbO_3_ samples was determined with a static method of a hanging drop by means of a ThetaLite optical tensiometer (Biolin Scientific, Espoo, Finland). It has been found that the surface tension of used Ag ink is equal to 39.8 × 10^−3^ N/m. The contact wetting angle of the sample surface was measured by the method of a sitting drop by means of recording the lateral profiles of the ink drops. These measurements were carried out for both rough and polished surfaces of the LiNbO_3_ samples. 

## 3. Results and Discussion

### 3.1. Morphology of the Printed Electrodes

The printed samples were annealed to obtain a homogeneous surface of the electrodes and remove the residual organic solvent from the ink. The effect of sintering temperature on the morphology of the printed electrodes for selection of the optimal sintering temperature regime was studied.

**Figure 4:**
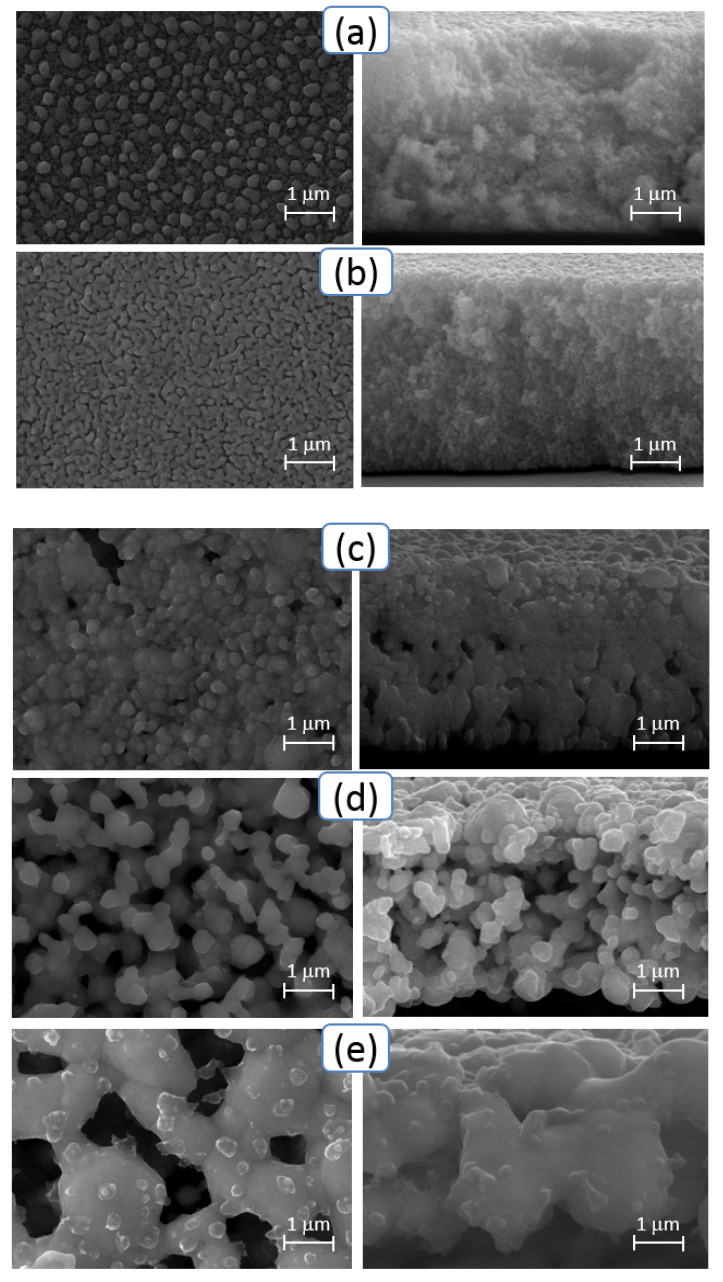
SEM images of the surface (left) and the cleavage (right) for the printed electrodes on Si samples obtained at the annealing temperatures of 100 °C (**a**), 200 °C (**b**), 300 °C (**c**), 400 °C (**d**), and 500 °C (**e**).

Figure 4 presents images of the surface (left) and the cleavage (right) for the electrodes on various Si samples obtained at the annealing temperatures of 100 °C (a), 200 °C (b), 300 °C (c), 400 °C (d), and 500 °C (e). As is known, the melting temperature of the material of nanoparticles substantially depends on their size. This is associated with a decrease in the energy of vacancy formation and, correspondingly, with an increase in their concentration with a decrease in the size of nanoparticles [34].

In the case of silver, the melting temperature of the bulk material is 961.8 °C and decreases to ~500 °C with a decrease in particle size to values of ~50 nm or less [35]. In this connection, for particles with an average size of 10 nm, a more significant decrease in the melting temperature can be predicted.

The increase in contrast in the SEM image allowed us to conclude that an increase in sintering temperature from 100 to 200 °C leads to the removal of organic solvent residues from the printed structure. With a further increase in sintering temperature to 300 °C and 400 °C, sintering of nanoparticles into ever larger clusters with subsequent formation of a continuous surface at 500 °C was observed. The micrographs of cleavages of samples were used to determine the thickness of printed structures. It was equal to 4 µm at the average for all samples.

### 3.2. Resistivity of the Printed Electrodes

**Figure 5:**
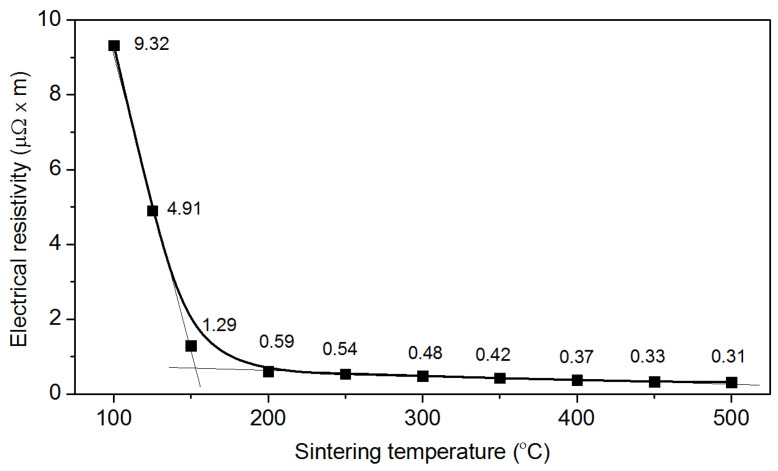
The dependence of electrical resistivity on sintering temperature.

In order to study the effect of sintering temperature on the resistivity of the printed electrodes deposited on Si, ten samples were made with a sintering temperature varying from 100 to 500 °C. In the measurement of the current-voltage characteristics, the knife-type gold probes were posed in such a way as to overlap the entire width of the printed electrode, and the distance between them was 10 mm. As a result, the linear current–voltage characteristics were obtained. Figure 5 shows the dependence of the electrode resistivity on the sintering temperature. 

The lowest resistivity (0.31 µΩ·m) was observed for a sample obtained at a sintering temperature of 500 °C. This was three times higher than the resistivity of the bulk Ag (0.104 µΩ·m [36]). The distinction was likely due to the inhomogeneous surface of the electrode (Figure 4e). Such distinction has been reported previously [8,22].

### 3.3. Contact Angle of Wetting

**Figure 6:**
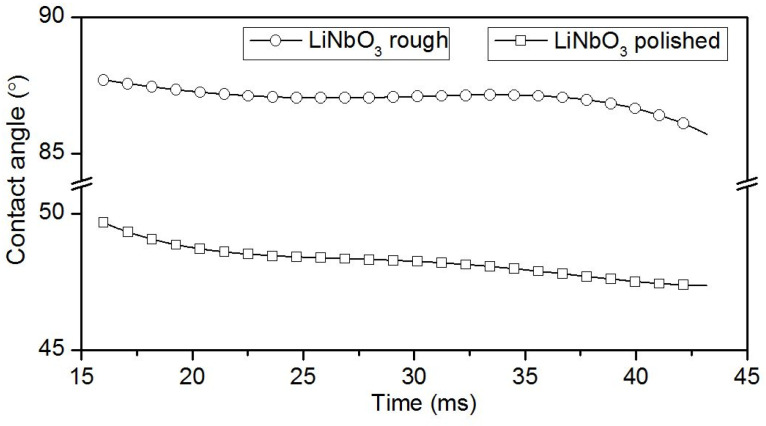
Time dependences of the contact wetting angle of Ag nano-ink deposited on a rough or polished surface of LiNbO_3_ sample during surface wetting.

**Figure 7:**
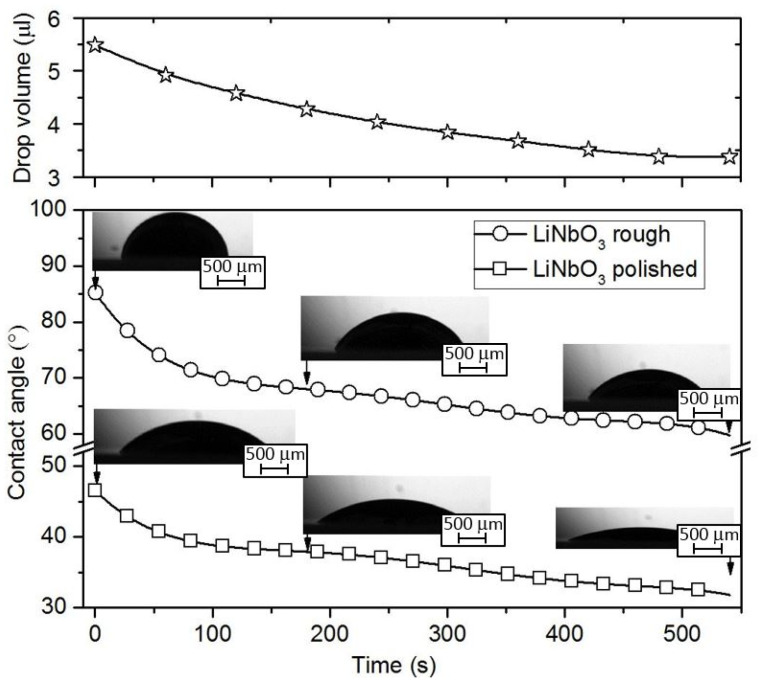
Time dependences of the contact wetting angle of Ag nano-ink deposited on a rough or polished surface of LiNbO_3_ sample during drop drying. Time dependence corresponding to the change in the drop volume on dry out is shown above.

In the printing electrode structures, an important aspect is the area of the ink drop on the surface of the substrate, since the step of moving the print head depends on this parameter. As is known, the area occupied by a drop on the surface of a substrate depends on the contact angle of wetting [37]. Figure 6 and Figure 7 show the experimentally obtained time dependences of the contact wetting angle of an Ag nano-ink drop deposited on a rough or polished surface of LiNbO_3_. Analysis of the time interval from 15 to 40 ms after ink deposition allowed us to study the process of wetting the substrates (Figure 6). The time interval from 1 to 540 s was used to study the dry out process of an Ag nano-ink drop (Figure 7). As expected, the contact wetting angle of a drop depends on the quality of the substrate surface. In the case of a rough surface, this angle is larger than in the case of a polished one. This is due to the distinction in the friction factor of different types of surfaces. Based on the data obtained, it is recommended to use the rough surface of LiNbO_3_ wafer to create electrode structures using conventional inkjet printing.

### 3.4. Piezoactive Acoustic Wave Excitation Using an Inkjet Printed Delay Line

**Figure 8:**
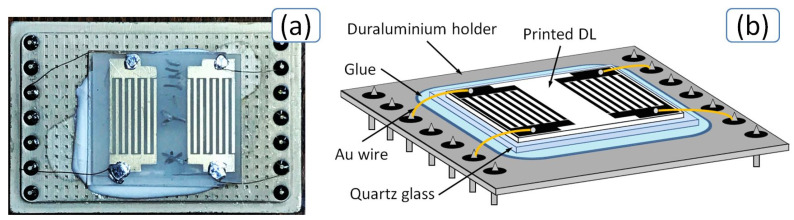
Optical microscopy image (**a**) and scheme (**b**) of the final chip with plate acoustic wave (PAW) device.

The PAW device was gold wires bonded by conductive glue. A photo (a) and scheme (b) of the final chip with produced delay line are presented in Figure 8. After calibration, the network analyzer was used to study S-parameters of the PAW device.

**Figure 9:**
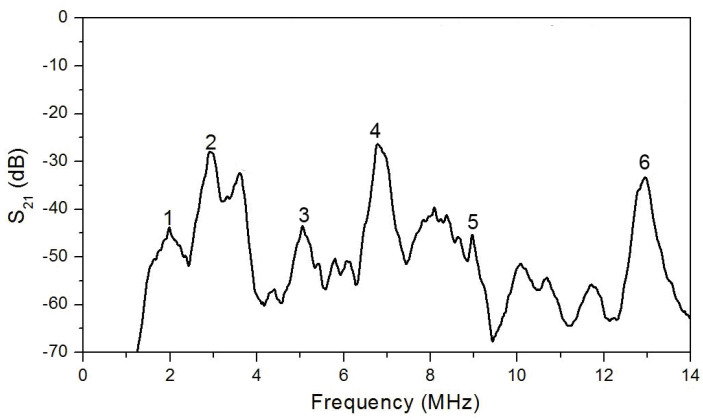
Frequency dependence of IDT transmission parameter S_21_ for produced delay line based on YX LiNbO_3_ plate.

Figure 9 shows the resulting transmission coefficients S_21_ in the frequency range of 1–14 MHz. It is seen that piezoelectric plate acoustic waves are successfully excited using printed IDTs and are characterized by enough low attenuation. 

### 3.5. Mathematical Modeling of the Inkjet Printed PAW Device by Using FEM Simulation

In mathematical modeling of the created delay line, a finite element modelling (FEM) simulation and a licensed software package Comsol Multiphysics 5.2 were used. The model was constructed in accordance with the geometry shown in Figure 3 and the data given in Table 1. The material constants of lithium niobate used in the calculation are given in Table 2 [38]. Data for bulk silver were used as material constants of the electrodes.

It is known that a YX LiNbO_3_ plate can propagate plate acoustic waves with high electromechanical coupling coefficients [39,40]. The theoretically estimated phase velocities (V_ph_) and the electromechanical coupling coefficients (*k*^2^) of the antisymmetric (A*_n_*), symmetric (S*_n_*) and the shear-horizontal (SH*_n_*) plate acoustic waves corresponding to experimentally observed peaks (Figure 9) are presented in Table 3. Here, *n* is an order of plate wave. The calculations were performed by using a conventional transfer matrix method [41].

*k*^2^ = 200 × (*V_ph_* − *V_phm_*)/*V_ph_*, (1)

where *V_ph_* and *V_phm_* are the phase velocities of the acoustic waves in electrically free and electrically shorted plates, respectively.

**Figure 10:**
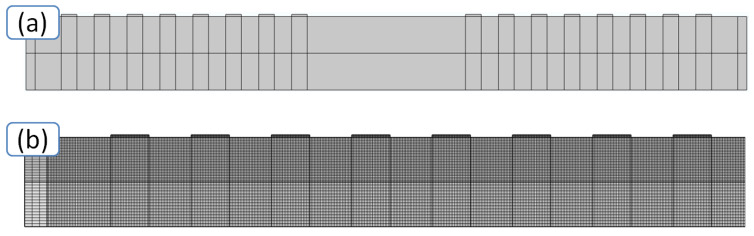
(**a**) Schematic model of a delay line, (**b**) enlarged mesh fragment.

The geometry of the IDT under study and a net used in the simulation are shown in Figure 10. On both surfaces of the plate, perfectly matched layers (PMLs) are located to prevent the reflections of excited waves. It was assumed that these absorbing layers were characterized by a quadratic frequency dependence of the attenuation. It was also assumed that the plate regions outside of the IDT were mechanically free, i.e., the mechanical stresses were equal to zero. In the area of the contact of the IDT fingers with the plate, the mechanical continuities of the mechanical displacements and stresses between the finger and the plate were used as the mechanical boundary conditions. The excitation of an acoustic wave was modeled by applying a variable electrical potential difference to the IDT fingers (Figure 10a). In the model, the IDT was represented by a set of equipotential rectangles, and the region under the electrodes was divided into the smaller elements. The linear size of these elements was equal to λ/50 (Figure 10b).

**Figure 11:**
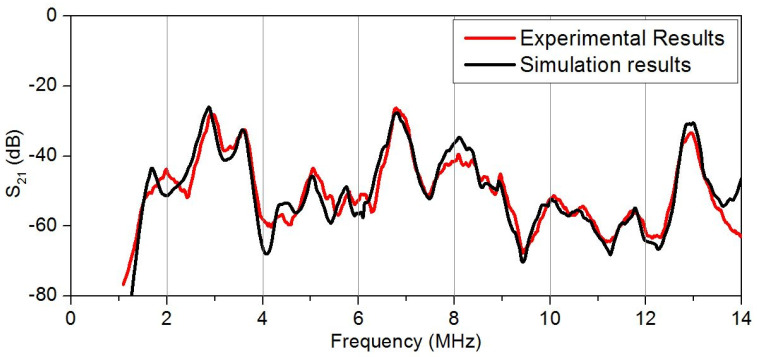
Comparison of simulation results with experimental data for produced delay line based on YX LiNbO3 plate.

A comparison of the theoretically calculated frequency dependence of parameter S_21_ for the created delay line with the experimentally obtained dependence is presented in Figure 11. It is possible to see the good coincide between theory and experimental results. 

## 4. Conclusions

The electrode structure of the delay line based on YX LiNbO_3_ for excitation of the plate acoustic waves was created by inkjet printing using silver nano ink. The thickness of the produced electrodes was equal to 4 µm. The lowest electrical resistivity of Ag strips was found to be 0.31 µΩ·m for a sample obtained at a sintering temperature of 500 °C. This is only about three times the value of the bulk silver. Contact wetting angle was shown to depend on the surface quality of the substrate. Based on obtained results, a rough surface of LiNbO_3_ wafer was recommended to use for production of electrode structure by means of inkjet printing. The FEM modeling of the excitation of acoustic waves by printed electrode structures showed a qualitative and a quantitative agreement with the experimental data. Thus, conventional inkjet printing can replace the complex photolithographic method for producing IDTs. The resulting electrode structures make it possible to efficiently excite piezoactive plate acoustic waves with large electromechanical coupling coefficients.

## Figures and Tables

**Table 1:** Geometric parameters of the printing layout of the developed delay lines. IDT: interdigital transducers.

Crystal Orientation, LiNbO_3_	The Number of Pairs of Electrodes	Strip Width (µm)	Strip Thickness (µm)	Distance Between Strips (µm)	Distance Between IDT (µm)	Aperture (µm)
Y-cut X-propagation directionYX	4	300	4	330	3030	7500

**Table 2:** Material constants of LiNbO_3_ crystal [38].

Elastic modulii, *C^E^_ij_* (10^10^ N/m^2^)						
*C^E^*_11_	*C^E^*_12_	*C^E^*_13_	*C^E^*_14_	*C^E^*_33_	*C^E^*_44_	*C^E^*_66_
20.3	5.73	7.52	0.85	24.24	5.95	7.28
**Piezoconstants, *e_ij_* (C/m^2^)**				**Dielectric permittivity, *ε^S^_ij_*/ε_0_**		**Density (kg/m^3^)**
*e*_15_	*e*_22_	*e*_31_	*e*_33_	*ε^S^*_11_	*ε^S^*_33_	*ρ*
3.83	2.37	0.23	1.3	44.3	27.9	4650

**Table 3:** Calculated *V_ph_* and *k*^2^ of the plate acoustic waves propagated in YX LiNbO_3_ plate.

Peak Number	Wave Type	*V_ph_* (m/s)	*k*^2^ (%)
1	A_0_	2467	6.0
2	SH_0_	4506	27.0
3	S_0_	6473	1.2
4	SH_1_	9034	10.8
5	A_1_	11,423	2.2
6	SH_2_	16,327	1.2
